# Loneliness Among Community-Dwelling Older Adults in Taiwan: Exploring Multi-Level Risk Factors

**DOI:** 10.1093/geroni/igaf122.304

**Published:** 2025-12-31

**Authors:** Honghui Pan, LiFan Liu, Su-I Hou

**Affiliations:** Vrije Universiteit Brussel, Brussels, Brussels Hoofdstedelijk Gewest, Belgium; National Cheng Kung University, Tainan City, Tainan, Taiwan (Republic of China); University of Central Florida, Orlando, Florida, United States

## Abstract

Loneliness has emerged as a significant societal issue in many Western countries, but limited research has explored loneliness among older adults in Taiwan, a rapidly aging society in East Asia. This study aimed to examine the prevalence and risk factors of loneliness among Taiwanese older adults during the COVID-19 pandemic. We analyzed secondary data from the University Responsibility dataset at National Cheng Kung University in southern Taiwan, including 530 adults aged 65 and above. Loneliness was measured using the 6-item De Jong Gierveld loneliness scale (2006), assessing emotional and social loneliness. Hierarchical multiple linear regression was applied to examine community-level (e.g., non-age-friendly environments, low social capital, low social support) and individual-level (e.g., poor living environment perception, low social participation) risk factors. Demographic factors such as age, gender, marital status, ethnicity, education, health, and income were also included. The results showed that 59.2% of participants experienced moderate loneliness, while 19.6% reported severe loneliness. Additionally, 36.5% faced emotional loneliness, and 58.4% social loneliness. Key community-level risk factors included low social capital (β=-0.341, P = 0.024) and low social participation (β=-0.365, P = 0.035). Individual-level risk factors included negative living environment perceptions (β=-0.700, P < 0.001), poor health (β=-0.452, P < 0.001), low income (β=-0.507, P = 0.026), and being married (β=-0.391, P = 0.037). These findings underscore the need for community-based interventions to enhance social capital and participation, and for promoting positive perceptions of living environments to reduce loneliness among older Taiwanese adults.

